# Major Congenital Malformations in Barbados: The Prevalence, the Pattern, and the Resulting Morbidity and Mortality

**DOI:** 10.1155/2014/651783

**Published:** 2014-04-06

**Authors:** Keerti Singh, Kandamaran Krishnamurthy, Camille Greaves, Latha Kandamaran, Anders L. Nielsen, Alok Kumar

**Affiliations:** ^1^Faculty of Medical Sciences, University of the West Indies, Cave Hill, BB23034, Barbados; ^2^The Queen Elizabeth Hospital, Martindales Road, Street Michael, BB14018, Barbados

## Abstract

*Objectives*. To study the prevalence and the pattern of major congenital malformations and its contribution to the overall perinatal morbidity and mortality. * Methods*. It is a retrospective population based study. It includes all major congenital malformations in newborns during 1993-2012. The data was collected from the birth register, the neonatal admission register and the individual patient records at the Queen Elizabeth Hospital where over 90% of deliveries take place and it is the only facility for the care of sick newborns in this country. * Results*. The overall prevalence of major congenital malformations among the live births was 59/10,000 live births and that among the stillbirths was 399/10,000 stillbirths. Circulatory system was the most commonly affected and accounted for 20% of all the major congenital malformations. Individually, Down syndrome (4.1/10, 000 live births) was the commonest major congenital malformation. There was a significant increase in the overall prevalence during the study period. Major congenital malformations were responsible for 14% of all neonatal death. * Conclusions*. Less than 1% of all live newborns have major congenital malformations with a preponderance of the malformations of the circulatory system. Major congenital malformations contribute significantly to the overall neonatal morbidity and mortality in this country.

## 1. Introduction

Congenital malformation is defined as “a permanent change produced by an intrinsic abnormality of development in a body structure during prenatal life” [1]. Reported prevalence of major congenital malformations in different population around the world has shown considerable variation and ranges from less than 1% to up to 8% [2–9]. The varying pattern and prevalence of congenital malformations over time or geographical location may reflect differing methods of detection and recording or true differences in frequency due to the complex interaction of known and unknown genetic and environmental factors including sociocultural, racial, and ethnic variables. For quite some time, congenital malformations have been reported to be a major cause of mortality and morbidity in children in the developed countries [10–15]. Contrary to the commonly held view that congenital disorders are not a public health issue in developing countries, in recent years, a number of developing countries are in fact experiencing an epidemiological transition, with significant declines in infant mortality rates, reduction of infections and malnutrition and a relative increase of morbidity and mortality due to congenital malformations [4, 8, 16–21]. There are numerous studies from the developed countries on the prevalence and the pattern of congenital malformation and their trends from well-designed multicenter surveillance programs [6, 8, 10–13, 15, 22]. However, epidemiological data on congenital malformations from long-term population-based studies originating from the developing countries is scanty [3, 5, 23, 24], and that from the Caribbean region is lacking altogether. The good epidemiological data on the prevalence rate and pattern of birth defects in a specific region gives an opportunity of identifying some etiological factors and can be useful for their prevention in this country and in the region.

Barbados is one of the English speaking Caribbean countries and has a total population of 250010 (2011 census), the majority of them being of African descent. Its gross domestic product (GDP) is US$ $23,700 (2011 est.) and its total (public plus private) expenditure on health as a percentage of GDP (2009) stands at 7% [25]. The infant mortality rate (2005) is 17.3 per 1000 live births [24]. It has a well-organized state run health care infrastructure with free health care for its citizens. There are polyclinics which serve as the primary health care delivery points and a single tertiary health care institution with specialized neonatal care unit where over 90% of deliveries take place. The captive population of this island state and the single centralized delivery and neonatal care facility provides good opportunity for a population based epidemiological study of congenital malformations. The purpose of the present study was to provide a descriptive overview of the epidemiology of congenital anomalies in Barbados and specifically to answer three questions. (1) What is the prevalence of congenital malformations? (2) Has there been a recent secular change in the prevalence of congenital malformations? (3) What pattern of congenital malformations is seen in this population? (4) What is the magnitude of contribution of congenital malformations to the overall perinatal morbidity and mortality?

## 2. Methods

This was a retrospective descriptive clinical audit of all the major congenital malformations in Barbados. It includes all live births and stillbirths during the period extending from 1993 to 2012. Both the spontaneous and induced abortions were excluded from the study due to the unavailability of good data. The main sources of data for this study were the delivery registry maintained in the department of obstetrics and the newborn registry at the neonatal care unit as well as the newborn hospital records at the Queen Elizabeth Hospital (QEH). The majority (over 90%) of the deliveries in Barbados took place at the Queen Elizabeth Hospital which has a well-equipped neonatal unit. Close to 100% of all pregnant women in Barbados receive antenatal care. The point of entry for the antenatal care for majority of the pregnant women is the state run polyclinics which are located all over the country and serve as the primary health care facility under the public health care network of this country. A minority of pregnant women also received their antenatal care either fully or partially through the private offices of the obstetricians or the general practitioners and these women have the choice of having their delivery at the QEH after referral or at the other private birthing center where less than 10% of all deliveries take place. Details of all the deliveries at the QEH are entered into the birth registry at the QEH. All babies born at the QEH are examined by the house staff in pediatrics with in the 24 hours of delivery. All babies detected to have a major congenital malformation are routinely transferred to the neonatal unit for further investigation, observation, and/or management. Normal babies are routinely discharged home at around 48 hours after delivery. Minority of the babies born outside of the QEH and who are found to have major congenital malformations are also transferred in to the NICU at the QEH for further care. All admissions to the neonatal unit are recorded in a special register maintained on the unit.

Live births and stillbirths (death in fetus after 28 completed weeks) with major congenital malformation were identified from the birth register and the neonatal register. Additional data on the live babies with major malformation were collected from their case record. Babies who were born at home or at the other birthing facility and had major congenital malformation and were transferred to the neonatal unit were also identified from the neonatal register. Data collection was performed by means of structured form which contained two parts. At first part, variables recorded were about maternal characters which were limited to the maternal age, geographic residence on the island, and the parity. The second part was about neonatal characters including date of birth, live birth or stillbirth, gestational age, sex, and pattern of CM, which were collected from the individual medical records. Neonatal outcomes in terms of death or discharge from the hospital were also recorded. Very few autopsy examinations were performed. Routine prenatal ultrasound imaging of the fetus was not available for the most part of the study period. Invasive prenatal diagnosis is not available in this country. Third part of data collection included data on the total live birth and the total number of newborns who required admission to the specialized neonatal unit as well as the neonatal death throughout the study period and these were collected from the birth register in the Obstetrics Department and the Neonatal Register on the Neonatal Unit at the QEH, respectively.

For the purpose of this report, congenital malformation was defined as a physical or anatomical abnormality detected at birth and classified according to categories listed in Chapter XVII: Congenital Malformations, Deformations, and Chromosomal Abnormalities (Q00-Q99) of the International Classification of Diseases (ICD10), 1997 [26]. Major congenital developmental disturbances are defined as structural defects of the body and/or organs that impair viability and require intervention [27, 28]. In some cases (particularly those with multiple anomalies), dysmorphologic evaluation and search for syndromes, features, and references were done by the neonatologist, using different sources: dysmorphology literature, “Smith's recognizable pattern of human malformation” [29]. Other variables selected for analysis included maternal age, gestational order, baby's sex, gestational age, and birth weight.

Prevalence of malformations was calculated in rates per ten thousand births according to the number of cases and noncases of malformed children. The prevalence of malformations in a number of different diagnostic subcategories is also reported separately. Thus, a child with trisomy 21, an atrioventricular septal defect, and duodenal atresia is counted once in the overall prevalence figures and once in the subcategory for malformations of the chromosomal defect but not separately in all the different subcategories such as chromosomal abnormality, circulatory system, and digestive system. All data was stored in Microsoft Access Database and Microsoft Excel was used for generation of graph and tables. Confidence intervals (95% CIs) were calculated for the total and live birth prevalence. The *χ* ^2^ test for trends was used to compare differences in prevalence over time. A *P* value of <0.05 was considered to be significant. Statistical analysis was performed using SPSS for Windows, v. 13.1. Necessary ethical approval was obtained from the Ethics Committee at the QEH as well as from the Institutional Review Board for Ethics in Human Subjects at the University of the West Indies (Cave Hill). All precaution was taken to protect the personal information of the patients and only the investigators had access to the data base.

## 3. Results

Over the twenty year study period (1993–2012), there were a total of 402 major congenital anomalies (376 among the live births + 26 among the stillbirths) recorded in the newborns from among the 64479 births (63827 live births + 652 stillbirths) in Barbados. There were 31658 live births with 149 cases of major malformations during the 1993 through 2002 period and 32169 live births with 227 cases of major malformations during the 2003 through 2012 period. Among the 376 live born babies with major malformation, there were 5 pairs (3%) of twins and the rest (97%) were from singleton pregnancy. The overall prevalence of major congenital malformations at birth was 62 (95% CI: 56 to 68) per 10,000 births over the study period. The prevalence of major congenital malformations among the live births was 59 (95% CI: 53 to 65) per 10,000 live births and the prevalence among the stillbirths was 399 (95% CI: 267 to 587) per 10,000 stillbirths.

The pattern of congenital malformation categorized according to the system involvement using the ICD-10 classification is shown in Table 1. Major malformations in the circulatory system accounted for 20% (96% CI = 16 to 25) of all the major malformations. Circulatory system malformation included congenital heart block −6, transposition of great vessels −6, hypoplastic left heart syndrome −5, pulmonary valve atresia −4, and tetralogy of fallot −3 cases. Other common systemic categories of malformations in order of frequency were those of the musculoskeletal system—16% (95% CI: 12 to 19), digestive system—13.1% (95% CI = 9 to 16), nervous system—10.9% (95% CI: 8 to 15), and chromosomal abnormality—9% (95% CI 6 to 12%). Overall, 31 (8%, 95% CI: 6 to 12%) babies had multiple major congenital malformations involving two or more systems with prevalence of 5/10,000 live births. The frequency of the individual major congenital malformations is shown in Table 2. Down syndrome (4.1/10,000 live births) was the commonest major congenital malformation followed by cleft lip and palate (4/10,000 live births).

The prevalence of major congenital malformation at birth by infant characteristics is shown in Table 3. Major congenital malformations were significantly more prevalent (OR = 0.3, 95% CI = 0.21 to 0.34) among the low birth weight babies (<2500 grams) when compared with the normal birth weight babies and large birth weight babies (*P* = 0.017) and among the preterm babies (<37 completed weeks of gestation) compared to the term babies (OR = 0.4, 95% CI = 0.32 to 0.51; Chi-Square, *P* = 0.0001). Males had significantly higher prevalence compared to females (OR = 1, 95% CI = 1. to 2; Chi-square, *P* = 0.006). The prevalence of major congenital malformation at birth was significantly (OR = 2, 95% CI = 1.5 to 2.4; Chi-square, *P* = 0.001) higher among the women in the age group ≥35 years when compared to those bellow 25 years and among those who were multigravida (OR = 4, 95% CI = 3 to 6; Chi-square *P* = 0.0001) compared to those who were primigravida. There was no statistically significant time trend noted when these maternal and infant variables for the period 1993 through 2002 were compared with those for the period 2003 through 2012.

**Figure 1:**
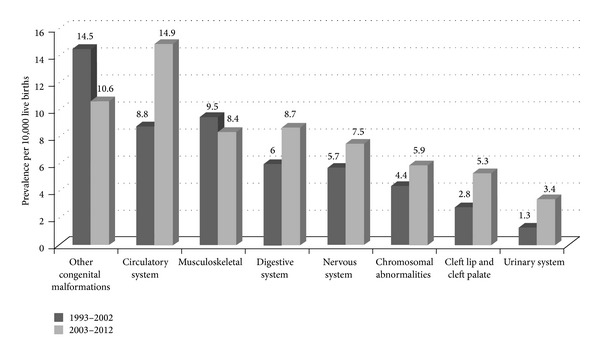
Pattern of congenital malformations in Barbados, 1993–2012.

There was a significant increase in the prevalence of the major congenital malformations from 47 (95% CI: 40 to 55) per 10,000 live births during the 1993–2002 to 71 (95% CI: 62 to 81) per 10, 000 live births during the 2003–2012 (*P* = 0.0001). Increasing secular trend was seen in the prevalence of the major malformations of the circulatory system, digestive system, and those of cleft lip and palate (Figure 1). The prevalence of the major malformations of the circulatory system increased from 9 per 10, 000 (95% CI = 6 to 13 per 10,000) during the 1993–2002 to 15 per 10,000 (95% CI = 11 to 20 per 10,000) during the 2003–2012 and those of the digestive system increased from 9 to 15, chromosomal abnormality increased from 4 to 6, and those cleft lip and palate increased from 3 to 5 per 10,000 live births during the corresponding period. However, none of these differences in the prevalence over time were statistically significant.

During the study period, 12004 (19%; 95% CI = 18.5% TO 19.1%) babies from among the 63827 live births required admission to the special neonatal unit. Overall, 3% (95% CI = 3% to 4%) of all newborn admissions to the specialized neonatal unit during the study period were for congenital malformations (Table 4). Proportion of all admissions to the neonatal care unit resulting from congenital malformations increased significantly (*P* < 0.0001) from 2.5% (95% CI = 2% to 3%) during the period 1993 through 2002 to 4% (95% CI = 3% to 4%) during the period 2003 through 2012. Mean duration of stay on the special neonatal unit for the term babies with major malformation was 11 days compared with 5 days for the term babies without major malformations. Over all, there were 627 (10/1000 live births) neonatal deaths from among 63827 live born babies. Neonatal mortality rate was 23.4/1000 among babies with major malformations and 9/1000 among babies without major malformation (OR = 0.308; 95% CI = 0.2 to 0.4 *P* < 0.0001). Major congenital malformation was responsible for 14% (95% CI = 11% to 17%) of all neonatal death and this was statistically significant (*P* = 0.0001). Proportion of neonatal deaths resulting from the major congenital malformation during the 1993−2002 period was 11% compared to 17% during the period 2003 through 2012. However, this was statistically not significant (*P* = 0.8).

## 4. Discussion

Prevalence studies of congenital anomalies are useful to establish baseline rates, to document changes over time, and to identify clues to aetiology. They are also important for planning and evaluating antenatal screening for congenital anomalies, particularly in high risk populations. The prevalence rates of major congenital malformations reported from around the world have shown large variations ranging from less than 100 to over 450 per 10,000 births [2–9, 14, 15, 30–32]. Much of the differences in the reported prevalence have probably resulted from the differences in the study design especially the data source, the length of observation, and the methods used for definition and categorization of the malformations. The population based data from a captive population with high uptake of antenatal care and a single hospital based delivery facility together with the definition and classification of congenital malformations based on the ICD10 in this report should provide good quality data suitable for easy international comparison.

The overall prevalence of major congenital malformation in this country during the study period was at 62 per thousand total births (including stillbirth after 28 completed weeks of gestations and live births) and 58 per 10,000 live births. Our prevalence rate is lower than those reported in many other population based studies in published literature which varies between 100 and 300 per 10,000 births [5, 23, 24, 33]. However, this difference is most likely due to the difference in the study design and our study compares well with the prevalence rate of congenital malformations from some other similarly designed population based studies using ICD10 classification that have reported similarly lower prevalence rates of less than 100 per 10, 000 births [3, 9, 32, 34]. It is interesting to note that the only other population based study from the Caribbean is from Cuba and has reported similarly low prevalence of congenital malformation at 47/10,000 live births [2].

In this study, congenital anomalies of the circulatory system (prevalence = 117 per 10,000 live births) was the most common malformation and accounted for 20% of all major CMs are seen during the early neonatal period in this population. This was followed by malformations of the musculoskeletal system (16% of all malformations) and digestive system (13% of all malformations). Once again, it is evident from the literature that there are large reported variations in pattern of congenital malformations involving different body systems in different populations around the world [3–9, 14–16]. A similar study from Saudi Arabia reported that the major congenital anomalies among all live births were mostly observed in the cardiovascular system (CVS), followed by musculoskeletal/limb [14]. In another study from Nigeria with similar ethnicity, the highest occurrence was in the skeletal system with 132 cases (29.4%) found, with an incidence of 1.03 per 1000 births [3].

Interpretation of birth defects occurrence trends is difficult because of the several factors that may influence reporting. These include ease, precision, and uniformity of diagnosis; classification, coding, and reporting; and the infant's age at the time the defect is usually recognized. Changes in the rates of defects readily apparent at birth, such as spina bifida and cleft lip and palate, are more likely to be actual changes than those reported for renal or heart defects, for example, because diagnosis of the latter defects requires more careful clinical examinations or special diagnostic techniques.

The present study demonstrates a significant increase in the prevalence of MCMs when the prevalence rate for the period 1993 to 2002 was compared with those from the period 2003-2012. Long-term studies from other countries have reported varying secular trends in the prevalence of CMs [7, 13, 22, 30, 31, 35]. Studies from UK and the USA have reported an overall decreasing trend and have attributed the decline to increasing prenatal diagnosis and termination of pregnancy in addition to the factors mentioned above [22, 30, 31]. EUROCAT surveillance reports have noted increasing trend in some of the member countries and decreasing trend in others [13, 35]. In addition to the true geographic variation, some of the differences could have been due to the method of ascertainment in the various registries. In our own study, although we cannot rule out the impact of the above mentioned factors such as changing case ascertainment methods in our data, we suspect that they are of minor importance given the relative methodological consistency in our data source, data collection, and analysis over time. Improving antenatal screening in the latter part of the period of reporting and better postnatal diagnosis may, however, have increased the ascertainment rate and therefore the prevalence of some CMs.

When the CMs of specific body system (ICD-10 class) were analyzed for trend, there was an increasing trend noted in the prevalence of CMs of circulatory system, digestive system, cleft lip and palate, chromosomal abnormality, and nervous system. There was a decreasing trend noted in the prevalence of musculoskeletal malformations and prevalence of the CMs of other systems remained unchanged over the 20 year study period. However, none of these increases in trends was statistically significant. However, the numbers for the malformations of the specific body system were small for any statistical power in the analysis. Similar observations have been made in other long-term studies from the developed countries [7, 13, 22, 30, 31, 35]. There were no long-term population based studies from the English speaking Caribbean region available for comparison.

Among the maternal characteristics that were studied for the risk of CMs, maternal age >35 years and gestational order >1 were both associated with higher risk. Similar observations have been made in other published studies [36, 37]. Prematurity (<37 completed weeks) and low birth weight (<2500 grams) were both associated with higher risk of CM in the newborn. Once again these are documented risk factors for congenital malformations [32, 36, 37].

Overall during the 20 year study period, major CMs were responsible for 3% of all admissions to the special neonatal care unit. However, there was a significant increase in the proportion of all admissions to the special neonatal unit during 2003 through 2012 when compared with the numbers during 1993 through 2002 (Table 4). The average stay of 15 days seen in this study was longer when compared to admissions from other causes (6 days) excluding prematurity. Babies with major CMs had a significantly higher risk of neonatal mortality compared to babies without major CMs. Overall, major CMs contributed significantly (*P* = 0.0001) to the neonatal deaths in this country and were responsible for over one-eighth of all neonatal deaths. Similar observations have been made in studies from some other developing countries [19, 20, 32, 38] and many developing countries prior to the widespread use of antenatal screening and elective medical termination of pregnancy in cases where major malformation was detected [11, 12, 27].

The major limitation of this study was retrospective nature of the study based on data derived from passive surveillance sources. This method may have compromised the case ascertainment and the prevalence may have been underreported in this study. Lack of routine autopsy study in cases of stillbirths may have also resulted in lower rate of detection of malformations among the stillbirths. Less than optimum use of antenatal and postnatal screening for congenital malformations during the earlier part of the reporting years could have been another source for lower than actual prevalence of major malformations in this country.

## 5. Conclusion

In conclusion, less than 1% of all live newborns have major congenital malformation which compares well with those reported in similarly designed recent studies from around the world. An increasing secular trend has been observed, but it could be a reflection of the better ascertainment of the major malformation in the recent years especially those of the circulatory system which was noted to be the most prevalent major malformation in this population. Major congenital malformation is becoming an increasing burden on the health care resources with close to 5% of all admissions to the neonatal unit and a higher than average unit stay from these cases. Also major CMs are contributing to over a tenth of the neonatal death in this country. Therefore, any measures undertaken for further reduction in perinatal mortality in this country will have to address the issue of CM. The first step in that direction would be active surveillance system for CM with setting up a CM register in this country.

## Figures and Tables

**Table 1:** Systemic classification of the major congenital malformations seen in the early neonatal period in Barbados, 1993–2012.

Class	Number	Percentage	Prevalence*	Confidence interval	ICD-class
Chromosomal Abnormalities	32	8.7	5	3 to 7	Q90–Q99
Circulatory system	77	20.4	12	10 to 15	Q20–Q28
Cleft lip and cleft palate	25	6.5	4	3 to 6	Q35–Q37
Digestive system	50	13.2	8	6 to 11	Q38–Q45
Genital system	13	3.5	2	1 to 4	Q50–Q56
Musculoskeletal	57	15.5	9	7 to 12	Q65–Q79
Nervous system	42	11.1	7	5 to 9	Q00–Q07
Other congenital malformations	56	15.1	9	9 to 12	Q80–Q89
Respiratory system	10	2.7	2	1 to 4	Q30–Q34
Urinary system	15	4.1	2	1 to 4	Q60–Q64

*Prevalence over the 20 years period, with 63827 live births, is expressed as number of Major Congenital Malformations per 10,000 live births.

**Table 2:** The frequency of individual major congenital malformation seen at birth in Barbados.

Congenital malformations	Number (%)	Prevalence per 10,000 live births
Down syndrome	26 (6.9%)	4.1 (3 to 6)
Cleft lip and palate	25 (6.6%)	3.9 (3 to 6)
Spina bifida	19 (5.1%)	3.0 (2 to5)
Hydrocephalus	17 (4.5%)	2.7 (2 to 5)
Exomphalos	16 (4.3%)	2.5 (2 to 5)
Small intestine obstruction	15 (4.0%)	2.4 (2 to 5)
Gastroschisis	14 (3.7%)	2.2 (1 to 4)
Tracheoesophageal fistula	12 (3.2%)	1.9 (1 to 4)
Ambiguous genitalia	12 (3.2%)	1.9 (1 to 4)

**Table 3:** Congenital malformations at birth by infant and maternal characteristics in Barbados, 1993–2012.

Selected demographic characteristics	Major congenital malformations	Total live births	Prevalence per 10,000
Infants gestational age	*N* = 370		
<37 weeks	89 (24%)	7213	123 (99 to 152)
37–40 weeks	281 (75%)	56614	50 (44 to 56)
Infants birth weight	*N* = 368		
<2500	104 (28%)	6047	172 (141 to 209)
2500–4000	252 (67%)	54901	46 (41 to 52)
>4000	12 (3%)	2879	42 (23 to 75)
Infants gender	*N* = 360		
Female	153 (42.5%)	31587	48 (41 to 56)
Male	207 (57.5%)	32040	65 (57 to 75)
Maternal age	*N* = 370		
15–24	108 (29.2%)	22932	47 (39 to 57)
25–34	175 (47.3%)	30576	57 (49 to 66)
35–44	87 (23.5%)	10192	85 (68 to 105)
Gestational order	*N* = 337		
Gravida 1	33 (9.8%)	19982	17 (12 to 24)
Gravida >2	304 (90.2%)	44310	69 (62 to 77)
Plurality of gestation	*N* = 376		
Singleton	371 (98.7%)	63582	58 (52 to 64)
Multiple gestation	5 (1.3%)	710	70 (26 to 173)

**Table 4:** Admission to the specialized neonatal unit and neonatal mortality from major congenital malformations in Barbados, 1993–2012.

Neonatal morbidity measures	1993–2012	1993–2002	2003–2012	Chi-square test
All neonatal admissions (proportion of all live births)	12004 (18.8%)	6023 (19%)	5981 (18.6%)	
Admissions from congenital malformations (proportion)	376 (3.1%, 95% CI = 2.8% to 3.5%)	149 (2.5%, 95% CI = 2.1% to 2.9%)	227 (3.8%, 95% CI = 3.3% to 4.3%)	*P* < 0.0001
All neonatal mortalities (neonatal mortality rate/1000 live birth)	627 (9.8)	296 (9.3)	331 (10.3)	
Neonatal mortality from congenital malformations (proportion)	88 (14.2, 95% CI = 11.4% to 17%)	33 (11.1%, 95% CI = 7.9% to 15.4%)	55 (16%, 95% CI = 12.9% to 21.1%)	*P* = 0.06

Total live birth	63827	31658	32169	
